# Epidemiology and Management of Intussusception Among Children Aged Under 2 Years in Gujarat, India

**DOI:** 10.1007/s12098-025-05806-1

**Published:** 2025-11-11

**Authors:** Nirkhi R. Shah, Bhadresh Vyas, Pankaj Buch, Pareshkumar A. Thakkar, Jaishri Ramji, Niket Patel, Rajesh Chudasama, Jaydeep Ganatra, Jayesh R. Solanki, Rakesh S. Joshi, Varsha Sudhir Chaudhary, Namrata Kharat, Anupama Machathi, Ragavi Lingam

**Affiliations:** 1 Department of Pediatric Surgery, B. J. Medical College, Ahmedabad, Gujarat, India; 2 Department of Pediatrics, M. P. Shah Government Medical College, Jamnagar, Gujarat, India; 3 Department of Pediatrics, PDU Medical College, Rajkot, Gujarat, India; 4 Department of Pediatrics, Government Medical College Baroda and S.S.G. Hospital, Vadodara, Gujarat, India; 5 Department of Community Medicine, PDU Medical College, Rajkot, Gujarat, India; 6 Department of Pediatric Surgery, PDU Medical College and Maa Sharda Hospital, Rajkot, Gujarat, India; 7 Department of Community Health, Christian Medical College, Vellore, Tamil Nadu, India

**Keywords:** Intussusception, Surveillance, Rotavirus vaccine, Gujarat

## Abstract

**Objectives:**

Some rotavirus vaccines are associated with an increased risk of intussusception (IS) in children. This study investigated the epidemiology of IS in children aged under 2 y in Gujarat, India.

**Methods:**

Hospital-based surveillance of IS cases was conducted from July 2019 to December 2022 at four hospitals in Gujarat. Children aged under 2 y who met the level 1 Brighton criteria were enrolled. Data were collected on their sociodemographic and clinical characteristics, clinical management, feeding patterns, and immunization history.

**Results:**

Overall, 213 children with IS were enrolled. Their median age was 8 (interquartile range, 6–13) mo and 62.9% were boys. No seasonal variation in the incidence of IS was observed. The most common presenting complaints were vomiting (82.6%), abdominal pain (76.1%), and bloody stools (53%). Most cases (56.3%) were managed *via* radiological reduction; 21.6% underwent exploratory laparotomy and intra-operative reduction; 10.3% underwent bowel resection; and 11.7% were treated using mixed modalities. Children admitted more than 48 h after the onset were significantly more likely to require surgical intervention (33% vs. 17.9%, *p* = 0.002) and have hospital stays > 5 d (59% vs. 35.2%, *p* < 0.001). In total, 13 of 169 children with documented rotavirus vaccination had received a dose of rotavirus vaccine within 21 d of the onset of symptoms.

**Conclusions:**

Ongoing surveillance is needed to establish whether vaccination with the oral pentavalent rotavirus vaccine, ROTASIIL, is associated with an increased risk of IS. Early case detection and referral, and increased use of safe and non-surgical methods of treatment need to be encouraged.

## Introduction

Intussusception (IS) is one of the major causes of intestinal obstruction in infancy and childhood [1]. IS is a condition in which the proximal intestinal segment (intussusceptum) telescopes or invaginates into the lumen of the distal bowel (intussuscipiens) [1].

The global incidence of IS is approximately 74 per 100,000 child-year [2]. The incidence among children in India varies from 17.7 cases per 100,000 child-year in North India to 254 cases per 100,000 child-year in South India [3, 4].

The majority of cases (75%) of IS occur in children aged under 2 y, of whom 40% are seen between 3 and 9 mo of age [5]. The incidence of IS is approximately three-fold higher in boys than in girls [2, 5] and is associated with lymphoid hyperplasia in infancy and viral infections, such as adenovirus infections, in early childhood. Natural rotavirus infection was previously thought to cause IS; however, recent studies have revealed no such association [3]. The ileocecal region is the most common site for IS [2, 4]. The most common presenting complaints are vomiting, abdominal pain, and passage of red “currant jelly” stools, and a palpable sausage-shaped mass in right side of abdomen is a common sign [6]. The death rate from IS is higher in developing countries than in the developed countries. Death may occur due to complications such as bowel ischemia, peritonitis, and septic shock. Delay in diagnosis and intervention contribute to increased morbidity and mortality [7].

The World Health Organization (WHO) recommends surveillance to document the potential changes and attributable risks of IS after the introduction of rotavirus vaccines (RVVs) in national immunization programs [8]. In Gujarat, the oral pentavalent rotavirus vaccine, ROTASIIL, was introduced on July 1, 2019 [9].

Given the limited data on the epidemiology of IS in western India, this study aimed to characterize the epidemiology, clinical presentation, and management of IS among children hospitalized in tertiary care government facilities in Gujarat.

## Material and Methods

This multicenter observational study was conducted over a 42-mo period, from July 2019 to December 2022, at four hospitals in Gujarat as part of a larger, multistate IS surveillance study [10].

The study was conducted at four tertiary care government hospitals in Gujarat: (1) B.J. Medical College, Civil Hospital in Ahmedabad, (2) M.P. Shah Government Medical College in Jamnagar, (3) Vadodara Medical College and S.S.G. Hospital in Vadodara, and (4) P.D.U. Medical College and Maa Sharda Childcare in Rajkot.

A description of the inclusion and exclusion criteria, data collection tools, and methods is provided in the introductory article in this supplement [10].

The study was approved by the Institutional Review Board of Christian Medical College, Vellore (approval number: 12242, dated September 25, 2019), which was the coordinating center, and the Institutional Ethics Committee of each participating institution in Gujarat (B.J. Medical College approval number: 07/2020-BJMC; M.P. Shah Government Medical College approval number: 132/04/2019-MPS GMC; Vadodara Medical College approval number: 19/02/2020 BMC; and P.D.U. Medical College approval number: 02/2021-PDU).

All statistical analyses were conducted using Stata version 16.1 [11]. The median and interquartile range (IQR) were calculated for continuous variables, and categorical variables were summarized using frequencies and percentages. The chi-square test was used to compare categorical data between groups, and two-tailed *p*-values < 0.05 were considered statistically significant.

## Results

From July 2019 and December 2022, 338 children were screened for IS at the four study sites, of whom 213 (63%) met the inclusion criteria and were enrolled. The IS case ratio was 1.36 per 1,000 pediatric and pediatric surgery admissions.

**Fig. 1:**
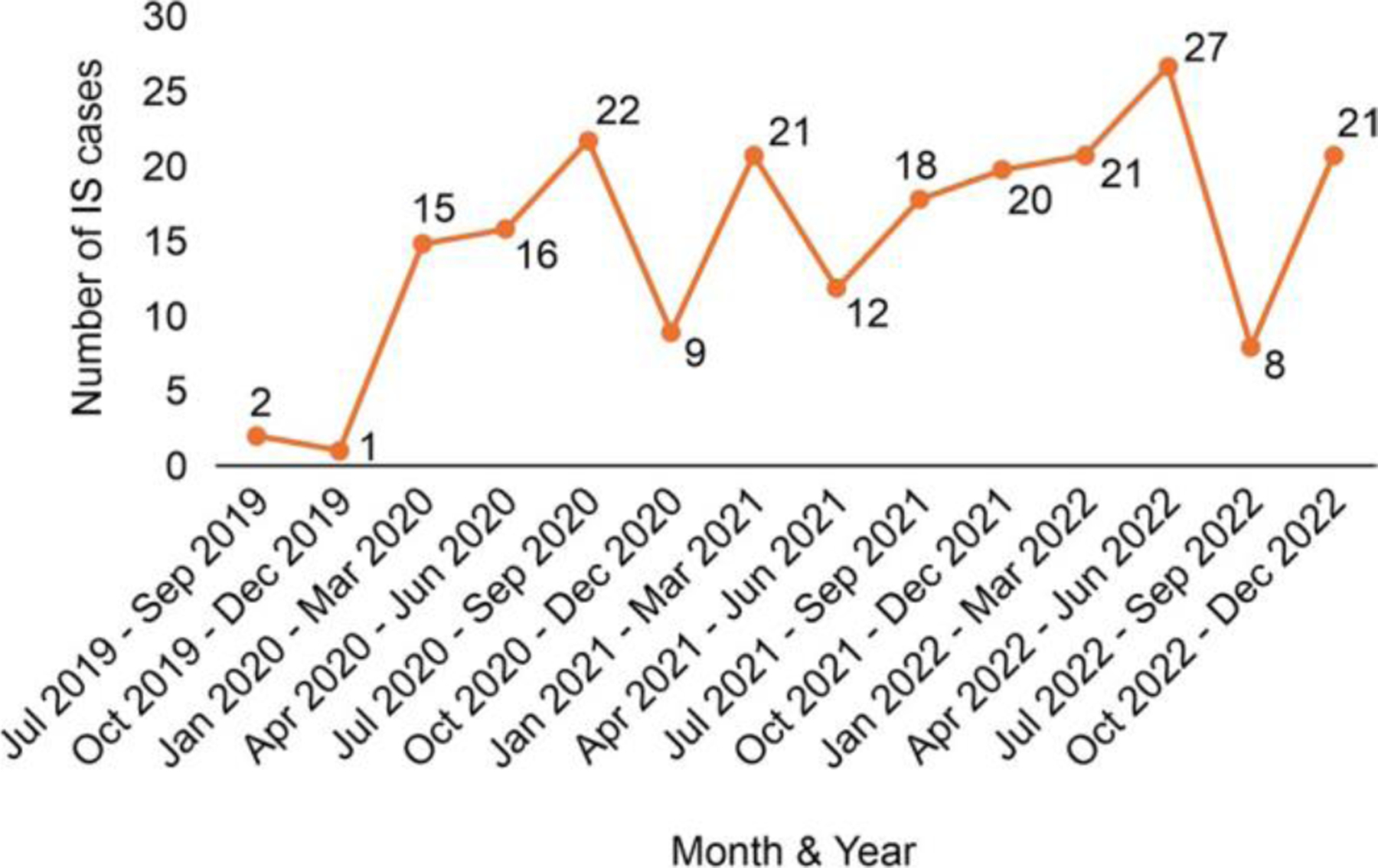
Number of cases of intussusception among children aged < 2 y hospitalized with intussusception in Gujarat from 2019 to 2022 by quarter (*N* = 213)

The median age at admission was 8 (IQR, 6–13) mo. Approximately half (53.6%) of the children were aged 5–9 mo, and 80.8% were younger than 15 mo (Table 1). The majority of children (62.9%) were boys; over half (52.1%) were referred from another facility (Table 1); and the majority (57.8%) were from families with lower socioeconomic status, and approximately half of the mothers (50.8%) had a primary school education or less (Table 1). The incidence of IS did not show any seasonal variation (Fig. 1). The most common presenting complaint was vomiting (82.6%), followed by abdominal pain (76.1%), and passage of blood-stained stool (53%). The ileocolic region was the most common site of IS (82.2%).

Of the 209 children (98.1%) with available vaccination records, 169 (80.9%) had received at least one dose of RVV. Of the 169 vaccinated children, 13 (7.8%) had received RVV within 21 d prior to the onset of IS. Among them, 3 had received the third dose, 1 had received the second dose within 7 d before onset, while 6 and 3 had received the third and second doses respectively 8–21 d before onset of symptoms (Table 1).

Of the 213 children, 120 (56.3%) had the IS reduced by non-surgical methods using either fluoroscope-guided pneumatic reduction or ultrasound-guided saline hydrostatic reduction, 46 (21.6%) underwent surgical reduction, 22 (10.3%) underwent bowel resection, and 25 (11.7%) were treated using mixed modalities (Table 2). Six children (2.8%) experienced a recurrence of IS before the age of 2 y.

A comparison of clinical manifestations by rotavirus vaccination status revealed that vomiting was significantly more common among vaccinated children than unvaccinated children (85.8% vs. 70.5%; *p* = 0.017) (Table 2).

Of the children who were admitted within 48 h of the onset of clinical manifestations, 63.6% underwent radiological reduction of IS. In children admitted > 48 h after the onset of symptoms, the rate of direct surgical intervention (reduction or resection) was almost double than that in children admitted within 48 h of onset of symptoms (51% vs. 25.9%), and the use of mixed modalities of treatment also increased in the first group of children (16% vs. 10.5%; *p* = 0.002) (Table 3).

The median period of hospitalization was 5 d (IQR, 2–8 d). A longer interval between onset and admission was associated with significantly longer hospital stay (*p* < 0.001) (Table 3). Among the 121 children who were discharged within 5 d of admission, 64.8% were admitted within 48 h of onset, whereas among the 92 children who required a hospital stay of > 5 d, 35.2% were admitted within 48 h of onset (*p* < 0.001) (Table 3).

Of the 203 children with breastfeeding histories available, 63 (32%) were exclusively breastfed and 134 (68%) had received breastmilk and supplementary feeding (Table 1).

## Discussion

This study documents the case load, age distribution, clinical features, and management of IS in Gujarat. The median age of admission in the Western Zone (11 mo) has been reported to be higher than in the whole of India (8 mo) [4, 12–14]. In this study, the incidence of IS was higher among boys than among girls, and the majority of children were aged 5–9 mo. These findings are consistent with other reports from India and globally [2, 4, 14–16].

Although the incidence of IS in other parts of India increased during the study period, the incidence in Gujarat remained stable [14, 16]. Contrary to some studies that reported an increased incidence during the summer and early monsoon season (April to July) [12, 14, 16, 17], no seasonal variation was observed in this study.

Vomiting, abdominal pain, and blood in stools were the most common clinical manifestations, consistent with other studies from the Western Zone of India and from other countries [4, 12, 14–18]. The commonest site of IS was ileocolic (82.2%), consistent with reports from other studies conducted in India and other countries (68–85.3%) [2, 4, 12, 17, 18]. The higher incidence of IS in the ileocolic region can be attributed to the increased lymphoid tissue in that region [19].

Over half of the patients were managed by radiological intervention, whereas 31.9% required surgical intervention, and 11.7% received a mixed modality of treatment. These results are comparable to those of other studies conducted in India in which similar treatment guidelines were followed [20]. Some multicenter studies have reported higher rates of surgical intervention because some centers do not offer radiological reduction [13]. Over half of the children in this study were from families with lower socioeconomic status with the mother having no formal education or primary education only. Poverty and lack of education may be associated with delays in seeking medical care, resulting in delay in transferring to an appropriate tertiary center.

Although the incidence of vomiting was significantly higher in vaccinated children than in unvaccinated children, no known mechanism can explain this finding; therefore, the association may be an incidental finding.

In this study, children were more likely to be successfully managed with radiological reduction when they presented early and the likelihood of surgical intervention and median length of hospital stay increased as the interval from onset to admission increased, consistent with the findings of other studies [12, 14].

Only 32% of the children were exclusively breastfed, which is lower than the national average (51%) [21, 22]. A shorter period of exclusive breastfeeding and early introduction of supplementary feeding may make children more susceptible to IS because weaning foods have been reported to increase the risk of IS [12]. However, this factor was not assessed in this study.

In this study, 13 of 169 children with documented rotavirus vaccination had received a second or third dose of RVV within 21 d of IS onset. As the age of administration of the third dose of RVV overlaps with the common age for IS [5], it was not possible to ascertain whether the risk of IS increased within the first 21 d after vaccination. This makes the association between IS and vaccination difficult to interpret. Hence, the Universal Immunization Programme recommends routine documentation of rotavirus vaccination in children with IS.

Strengths of this study include the enrollment of children from multiple hospitals in different parts of Gujarat, adherence to fixed protocols and standard case definitions, and use of vaccination records to confirm rotavirus vaccination status.

This study also has some limitations. It was a hospital-based surveillance study that only included children who were admitted to a tertiary-level government hospital with IS. Therefore, the findings may not be generalizable to all children in Gujarat. Patients treated at other public or private facilities were not included, which may have led to an underestimation of the disease burden.

## Conclusions

In this study, IS was most common in male infants of lower socioeconomic status, with no seasonal variation. Over half of the children were treated with radiological reduction. The probability of requiring surgical intervention and the duration of hospital stay increased as the interval between onset and hospital admission increased. To minimize morbidity and mortality, ongoing surveillance is needed to assess whether administration of the oral pentavalent rotavirus vaccine, ROTASIIL, is associated with a short-term increase in the risk of IS, improve case detection, early referral, and access to adequate medical management with safe, non-surgical methods.

## Figures and Tables

**Table 1:** Sociodemographic characteristics, and rotavirus vaccination status in children aged < 2 y hospitalized with intussusception in Gujarat between 2019 and 2022 (*N* = 213)

Characteristic	Number of cases, *N* = 213	Percentage
Age (mo)		
0–4	22	10.3
5–9	114	53.6
10–14	36	16.9
15–19	25	11.7
20–23	16	7.5
Sex		
Male	134	62.9
Female	79	37.1
Type of admission		
Transferred	111	52.1
Direct	102	47.9
Socioeconomic status of the family (tertile)		
Lower	123	57.8
Middle	84	39.4
Upper	6	2.8
Maternal education		
No formal education	17	8.6
Primary (up to standard 5)	84	42.2
Secondary and higher secondary (standards 6–12)	56	28.1
University and above	42	21.1
Rotavirus vaccination status (*n* = 209)^a^		
Unvaccinated	40	19.1
Vaccinated with at least one dose	169	80.9
Breastfeeding practices (*n* = 197)		
Exclusive breastfeeding	63	32
Mixed feeding (breastfeeding and supplementary feeding)	134	68
Time of intussusception since last rotavirus vaccination		
*Dose 1*		
1–7 d	0	0
8–21 d	0	0
> 21 d	168	78.9
*Dose 2*		
1–7 d	1	0.5
8–21 d	3	1.4
> 21 d	153	71.8
*Dose 3*		
1–7 d	3	1.4
8–21 d	6	2.8
> 21 d	132	62.0

a 4 children did not have available vaccination records

**Table 2:** Comparison of clinical course and management of intussusception among children aged < 2 y hospitalized with intussusception in Gujarat from 2019 to 2022 (*N* = 213)

Variable and category	All *N* = 213 *n* (%)	Unvaccinated *N* = 44 *n* (%)	Vaccinated^a^ *N* = 169 *n* (%)	*p*
Vomiting (*N* = 213)				**0.017**
Yes	176 (82.6)	31 (70.5)	145 (85.8)	
No	37 (17.4)	13 (29.5)	24 (14.2)	
Abdominal pain (*N* = 207)				0.329
Yes	162 (76.1)	36 (83.7)	126 (76.8)	
No	45 (21.1)	7 (16.3)	38 (23.2)	
Blood in stools (*N* = 212)				0.753
Yes	113 (53)	22 (51.2)	91 (53.8)	
No	99 (46.5)	21 (48.8)	78 (46.2)	
Diarrhea (*N* = 210)				0.455
Yes	65 (30.5)	11 (26.2)	54 (32.1)	
No	145 (68.1)	31 (73.8)	114 (67.9)	
Fever (*N* = 213)				0.968
Yes	78 (36.6)	16 (36.4)	62 (36.7)	
No	135 (63.4)	28 (63.6)	107 (63.3)	
Constipation (*N* = 210)				0.162
Yes	23 (10.9)	7 (17.1)	16 (9.5)	
No	187 (89)	34 (82.9)	153 (90.5)	
Management of intussusception (*N* = 213)				0.040^b^
Radiological reduction	120 (56.3)	23 (52.3)	97 (57.4)	
Surgical reduction	46 (21.6)	14 (31.8)	32 (18.9)	
Resection	22 (10.3)	6 (13.6)	16 (9.5)	
Mixed^c^	25 (11.7)	1 (2.3)	24 (14.2)	

When calculating *p* values, records with unknown results were excluded

a “Vaccinated” refers to children who have taken at least 1 dose of RVV

b Calculated using Fisher’s exact test

c Mixed management included 17 cases managed by radiological and surgical reduction; 2 cases managed by radiological reduction and resection; 2 cases managed by surgical reduction and resection; and 4 cases managed by radiological and surgical reduction and resection

**Table 3:** Management and length of hospital stay (d) according to the interval between onset of intussusception and admission (d) (*N* = 213)

	All^a^ (*N* = 213)	Time from onset to admission		*p*
		≤ 48 h^a^ (*N* = 162)	> 48 h^a^ (*N* = 51)	
Management, *n* (%)				0.002^b^
Radiological reduction	120 (56.3)	103 (63.6)	17 (33)	
Surgery	46 (21.6)	29 (17.9)	17 (33)	
Resection	22 (10.3)	13 (8.0)	9 (18)	
Mixed	25 (11.7)	17 (10.5)	8 (16)	
LOS (d), median (IQR)	5 (2–8)	4 (2–7)	7 (5–9)	< 0.001^b^
LOS (d), *n* (%)				< 0.001^c^
≤ 5	121 (56.8)	105 (64.8)	16 (31.4)	
> 5	92 (44.6)	57 (35.2)	35 (68.6)	

a Values are expressed *n* (column %) or median and interquartile range (IQR)

b Calculated using the Mann–Whitney U-test

c Calculated using the chi-square test

*IQR* Interquartile range; *LOS* Length of stay
